# Is waist-calf circumference ratio associated with frailty in older adults? Findings from a cohort study

**DOI:** 10.1186/s12877-023-04182-9

**Published:** 2023-08-15

**Authors:** Miao Dai, Quhong Song, Jirong Yue, Taiping Lin, Wei Jie, Xiang Wang, Ning Ge

**Affiliations:** 1 Department of Geriatrics, Jiujiang First People’s Hospital, Jiujiang, 332000 Jiangxi China; 2 Department of Geriatrics and National Clinical Research Center for Geriatrics, West China Hospital of Sichuan University, Chengdu, 610041 Sichuan China; 3 Department of Cardiology, Jiujiang First People’s Hospital, Jiujiang, 332000 Jiangxi China

**Keywords:** Waist-calf circumference ratio, Waist circumference, Calf circumference, Frailty, Older adult

## Abstract

**Background:**

The waist-calf circumference ratio (WCR) has been suggested as a potential indicator of visceral adiposity. Nevertheless, the relationship between WCR and the risk of frailty remains unclear. Therefore, our study aimed to investigate the association between WCR and longitudinal changes in WCR with frailty risk in older adults.

**Methods:**

We included 2359 participants aged ≥ 65 years without frailty (frailty index [FI] ≤ 0.21) from the Chinese Longitudinal Healthy Longevity Survey in the 2014 wave. The follow-up was conducted in 2018. We investigated the relationship of WCR, waist circumference (WC), and calf circumference (CC) with frailty using both the Cox proportional hazards model and the generalized estimating equation (GEE).

**Results:**

During a median follow-up of 4.0 years, 668 (28.2%) frailty occurred. Those with higher WCR and WC had a significantly increased risk of frailty (fifth quintile compared with first quintile: hazard ratio [HR] = 1.59, 95% confidence interval [CI] 1.24–2.04 for WCR; HR = 1.69, 95% CI 1.27–2.24 for WC), whereas those in the fourth quintile of CC had a lower likelihood of developing frailty compared to those in the first quintile (HR = 0.67, 95% CI 0.50–0.89). Interaction analyses showed that the effects of WCR on frailty were more pronounced in females (P-interaction = 0.016). GEE analyses revealed that increased WCR and WC were associated with a higher risk of frailty (odds ratio [OR] = 1.74, 95% CI 1.43–2.12 for WCR; OR = 1.03, 95% CI 1.02–1.04 for WC), while CC showed opposite results (OR = 0.95, 95% CI 0.93–0.97).

**Conclusions:**

A higher WCR and WC, as well as a lower CC, were significantly associated with higher frailty. Of these measures, WCR demonstrated the strongest association with frailty, suggesting that having a combination of high central fat and low lean body mass may increase the risk of developing frailty.

**Supplementary Information:**

The online version contains supplementary material available at 10.1186/s12877-023-04182-9.

## Background

As a geriatric syndrome, frailty is characterized by an increased vulnerability to minor stressors and decreased physiological reserve, which is common among older people [1]. It is suggested that the prevalence of frailty in community-dwelling older adults (65 years and older) is about 11% [2]. Worldwide, approximately 4.3% of adults over the age of 60 develop frailty annually [3]. Frailty can result in an inability to cope with stressful events and may lead to adverse outcomes such as falls, disability, delirium, hospitalization, institutionalization, and even mortality [4]. Therefore, identifying risk factors for frailty is crucial to the development of effective interventions aimed at preventing and treating this condition.

Obesity is a well-known risk factor for frailty [5, 6]. Body mass index (BMI) is commonly used to measure general obesity [7], while waist circumference (WC) is used to measure abdominal or central obesity [8]. However, aging can have negative effects on functional status, leading to a loss of muscle mass and an increase in fat mass [9], which may cause overall body weight and BMI to remain relatively unchanged. Given the opposing effects of abdominal fat and leg lean mass on disease risk, central fat and loss of fat-free mass may be more influential than BMI in determining health risks associated with obesity in older adults [10]. WC and calf circumference (CC) are simple and non-invasive measures that have been proposed as markers of adiposity and muscle mass, respectively, and have been used to assess frailty [11, 12]. The waist-calf circumference ratio (WCR) is a new index that combines both measures, serving as an effective method for evaluating the imbalance between abdominal fat and leg muscle mass. WCR has been proposed as a reliable predictor of cognitive impairment [13], carotid atherosclerosis [14], and health-related quality of life [15], and has been shown to be a superior predictor of health outcomes compared to BMI, WC, or CC alone. While several studies have investigated the relationship between anthropometric measures and frailty, few have focused on the WCR.

This study aims to fill this knowledge gap by analyzing the associations of anthropometric measures (WCR, WC, and CC) and their longitudinal changes over time with frailty in older adults using data from the Chinese Longitudinal Healthy Longevity Survey (CLHLS).

## Methods

### Study design and participants

The CLHLS is an ongoing prospective cohort study that aims to investigate the factors that influence healthy longevity among older adults in China. It is being conducted in 22 out of 31 provinces in the country. After the baseline wave in 1998, participants who were still alive have been followed up face-to-face every two to four years. The CLHLS uses a mixed longitudinal design, with approximately one-third of the participants in each wave being from the previous wave, while the rest are recruits. An in-depth description of the CLHLS is available elsewhere [16].

The baseline data for this study was taken from the 2014 wave of the CLHLS, as there were numerous missing values in CC in previous waves. The follow-up was conducted in 2018. Among 7192 older adults, we excluded the following participants: those aged < 65 years old (n = 85), those who lost to follow-up (n = 1511), missing data on frailty index (FI) at baseline and follow-up (n = 249), missing data on CC or WC at baseline (n = 288), CC or WC values which lay 1.5 interquartile ranges (IQRs) above the third quartile or 1.5 IQRs below the first quartile at baseline(n = 249), frailty at baseline (n = 1755), and death during follow-up (n = 696). Finally, a total of 2359 participants without frailty at baseline were included to analyze the association of WCR, WC, and CC with incident frailty. To analyze the association between longitudinal changes in anthropometric measures (WCR, WC, and CC) over time and frailty, we further excluded participants with missing data on CC or WC at follow-up (n = 53) and those with CC or WC values which lay 1.5 IQRs above the third quartile or 1.5 IQRs below the first quartile at follow-up (n = 88). We excluded observations that lay 1.5 IQRs above the third quartile or 1.5 IQRs below the first quartile of WC and CC to eliminate the effects of very sparse data. A detailed description of the inclusion and exclusion process is provided in Supplementary Fig. 1.

### Anthropometric assessment

The staff members conducted anthropometric measurements using standardized procedures. To measure WC, they located the midpoint between the lower ribs and the iliac crest and ensured that the tape measure was snug against the skin. The measurement was taken at the end of a normal expiration. CC was measured at its widest point with minimal pressure to avoid compressing the subcutaneous tissue. WCR was calculated by dividing the WC measurement (in cm) by the CC measurement (in cm).

### Outcome assessment

Frailty status was measured using the FI, a widely used tool [17]. According to Rockwood and colleagues [18], the variables selected for constructing a FI must fulfill the following criteria: (1) be health-related, (2) be associated with age, and (3) neither be excessively common nor excessively uncommon. To achieve adequate predictive accuracy for adverse outcomes, an index comprising 30–40 variables is considered sufficient. To construct the FI, we used 40 items following a standardized procedure described by Rockwood and colleagues [18]. These items included self-rated and interviewer-rated health, psychological characteristics, cognitive function, disability in daily activities, functional limitations, hearing and vision abilities, and chronic diseases (Supplementary Table 1) [19]. Health deficits were coded as a binary variable, with 1 indicating the presence of deficits and 0 indicating the absence. An ordered classification variable was also used, with scores ranging from 0 to 1. For example, vision loss was scored based on the level of impairment: can see and distinguish = 0, can see only = 0.5, and can’t see or blind = 1. A score of 2 was assigned to respondents who were bedridden or had more than one serious illness in the past two years. “Serious illness” was defined as a medical condition that significantly impacts a person’s health and daily functioning, often requiring intensive medical intervention and prolonged treatment. The FI was calculated by summing up the scores of all variables and dividing them by the total number of variables considered, resulting in a score ranging from 0 to 1. Frailty was defined as an FI value > 0.21, as applied in previous studies [20, 21].

### Covariates assessment

The baseline survey utilized a standardized and structured questionnaire to gather information on sociodemographic characteristics and health behaviors, including age, sex (male or female), education (0 years or 1 year or more), residence (rural or urban), living arrangement [living with family member(s) or others (living alone or in an institution)], marital status [married or others (divorced, widowed, or never married)], economic status (independence or dependence), smoking status (never, current, or former), drinking status (never, current, or former), regular exercise (never, current, or former), sleep time (< 6 h, 6 to 9 h, or ≥ 9 h), body mass index (BMI). BMI was calculated using weight (in kg) divided by height (in meters squared). BMI categories included underweight (BMI < 18.5 kg/m^2^), normal weight (18.5–24 kg/m^2^), overweight (24–28 kg/m^2^), and obese (≥ 28 kg/m^2^) [22].

### Statistical analysis

The presence of missing data may reduce the statistical power of a study and introduce bias into estimates of the relationship. To address this issue, we employed the multivariate imputation by chained equations method to conduct multiple imputation for missing data (ranging from 0.4 to 2.2%, Supplementary Table 2). Baseline characteristics were reported as means (± SD) for continuous variables and as percentages for categorical variables.

The ability of anthropometric measures (WCR, WC, CC, and BMI) to predict frailty was evaluated using receiver operating characteristic (ROC) curves, and their performances were compared using the Delong test. The optimal cut-point value for each parameter to predict frailty was determined by the highest Youden index (sensitivity + specificity − 1). Participants were categorized into five groups based on quintiles of their anthropometric measures (WCR, WC, and CC), or into two groups according to the optimal cut-point value. We utilized Cox proportional hazards models to calculate hazard ratios (HRs) and 95% confidence intervals (95% CIs) to assess the association between anthropometric measures and frailty. We assessed the proportional hazard assumption using Schoenfeld residuals and found no potential violations. To adjust for potential confounding factors, we constructed multivariable-adjusted models with baseline age, sex, marital status, education, residence, living arrangement, economic status, smoking status, drinking status, regular exercise status, sleep time, BMI, and FI. To further explore the independent effects of anthropometric measures on frailty, we adjusted for WC in the assessment of CC, and vice versa. We conducted a linear trend test by treating WCR, WC, or CC quintiles as continuous variables based on the median values of each quintile. We also estimated the crude incidence rate (per 1000 person-years) of frailty across categories of anthropometric measures. To examine the dose-response relationship between changes in anthropometric measurements and frailty risk, we conducted a restricted cubic spline analysis with four knots at the 5th, 35th, 65th, and 95th percentiles of changes in anthropometric measurements. We used the 50th percentile of WCR (2.62), WC (82.00 cm), or CC (31.00 cm) as the reference value. To examine the associations between longitudinal changes in anthropometric measures and frailty, we employed a repeated measures logistic regression model fitted using generalized estimating equations (GEE) with an exchangeable correlation structure. The data collection process involved obtaining anthropometric measures and covariates at two specific time points: the baseline in the 2014 wave and the follow-up in the 2018 wave. The dependent variable of the GEE model was the presence or absence of frailty, and the independent variables were the longitudinal changes in the anthropometric measures (WC, CC, WCR). The odds ratios (ORs) indicated the magnitude of the association between longitudinal changes in anthropometric measures and the risk of frailty while considering the influence of covariates over time. We conducted stratified and interaction analyses based on anthropometric measures (WCR, WC, and CC) and participant characteristics (age, sex, marital status, regular exercise, and BMI). Additionally, we performed sensitivity analyses using only complete cases and assessed the potential impact of the multiple imputation approach on our findings.

We conducted all analyses using R statistical software version 4.1.3 (R Foundation for Statistical Computing), and we used a two-tailed P value < 0.05 as the significance threshold.

## Results

### Basic characteristics of participants

Out of the 2359 participants, 52.7% were men, and their mean (SD) age was 80.0 (8.2) years. The mean (SD) of WCR was 2.66 (0.40). As shown in Table 1, the participants with higher WCR tended to be older, be women, be unmarried (either divorced, widowed, or unmarried), live alone or live in an institution, be illiterate, and be financially dependent. Additionally, those with higher WCR were more likely to be nonsmokers and nondrinkers, have a lower CC and BMI, and have a higher FI and WC.

**Table 1:** Population characteristics by quintiles of waist-calf circumference ratio

Characteristics	Quintile 1	Quintile 2	Quintile 3	Quintile 4	Quintile 5	P value
No. of participants	473 (20.1)	471 (20.0)	478 (20.3)	466 (19.8)	471 (20.0)	
Age (year), mean (SD)	78.52 + 7.73	78.44 + 7.46	80.04 + 8.25	80.74 + 8.12	82.45 + 8.78	< 0.001
Female, no. (%)	170 (35.9)	183 (38.9)	210 (43.9)	232 (49.8)	320 (67.9)	< 0.001
Living with family member(s), no. (%)	376 (79.5)	374 (79.4)	386 (80.8)	355 (76.2)	345 (73.2)	0.036
Married, no. (%)	285 (60.3)	299 (63.5)	280 (58.6)	237 (50.9)	215 (45.6)	< 0.001
Urban area, no. (%)	195 (41.2)	179 (38.0)	211 (44.1)	199 (42.7)	203 (43.1)	0.353
Education (year), no. (%)						< 0.001
0	191 (40.4)	184 (39.1)	200 (41.8)	218 (46.8)	282 (59.9)	
≥1	282 (59.6)	287 (60.9)	278 (58.2)	248 (53.2)	189 (40.1)	
Smoking status, no. (%)						< 0.001
Never	289 (61.1)	305 (64.8)	309 (64.6)	327 (70.2)	375 (79.6)	
Current	118 (24.9)	100 (21.2)	112 (23.4)	86 (18.5)	64 (13.6)	
Former	66 (14.0)	66 (14.0)	57 (11.9)	53 (11.4)	32 (6.8)	
Drinking status, no. (%)						0.001
Never	314 (66.4)	321 (68.2)	336 (70.3)	334 (71.7)	374 (79.4)	
Current	112 (23.7)	99 (21.0)	100 (20.9)	88 (18.9)	72 (15.3)	
Former	47 (9.9)	51 (10.8)	42 (8.8)	44 (9.4)	25 (5.3)	
Regular exercise, no. (%)						0.166
Never	306 (64.7)	290 (61.6)	295 (61.7)	304 (65.2)	325 (69.0)	
Current	157 (33.2)	171 (36.3)	170 (35.6)	157 (33.7)	134 (28.5)	
Former	10 (2.1)	10 (2.1)	13 (2.7)	5 (1.1)	12 (2.5)	
Economic independence, no. (%)	201 (42.5)	193 (41.0)	157 (32.8)	132 (28.3)	119 (25.3)	< 0.001
Sleep time (h), no. (%)						0.056
<6	71 (15.0)	67 (14.2)	63 (13.2)	58 (12.4)	87 (18.5)	
6–9	286 (60.5)	301 (63.9)	280 (58.6)	286 (61.4)	285 (60.5)	
≥9	116 (24.5)	103 (21.9)	135 (28.2)	122 (26.2)	99 (21.0)	
Frailty index, mean (SD)	0.09 + 0.05	0.09 + 0.05	0.10 + 0.05	0.10 + 0.05	0.11 + 0.05	< 0.001
Waist circumference (cm), mean (SD)	75.13 + 7.89	80.97 + 9.08	83.27 + 8.69	86.08 + 8.95	86.04 + 9.84	< 0.001
Calf circumference (cm), mean (SD)	34.63 + 3.87	32.97 + 3.68	31.82 + 3.37	30.82 + 3.28	26.80 + 4.04	< 0.001
BMI (kg/m^2^), no. (%)						< 0.001
Underweight (< 18.5)	71 (15.0)	54 (11.5)	50 (10.5)	54 (11.6)	81 (17.2)	
Normal (18.5–24)	301 (63.6)	278 (59.0)	294 (61.5)	232 (49.8)	259 (55.0)	
Overweight (24–28)	87 (18.4)	108 (22.9)	107 (22.4)	147 (31.5)	102 (21.7)	
Obese (≥ 28)	14 (3.0)	31 (6.6)	27 (5.6)	33 (7.1)	29 (6.2)	

*BMI* Body Mass Index

Notes: Differences in characteristics were compared using the χ^2^ test for categorical variables and ANOVA for continuous variables

### Association of anthropometric measures with incident frailty

**Fig. 1:**
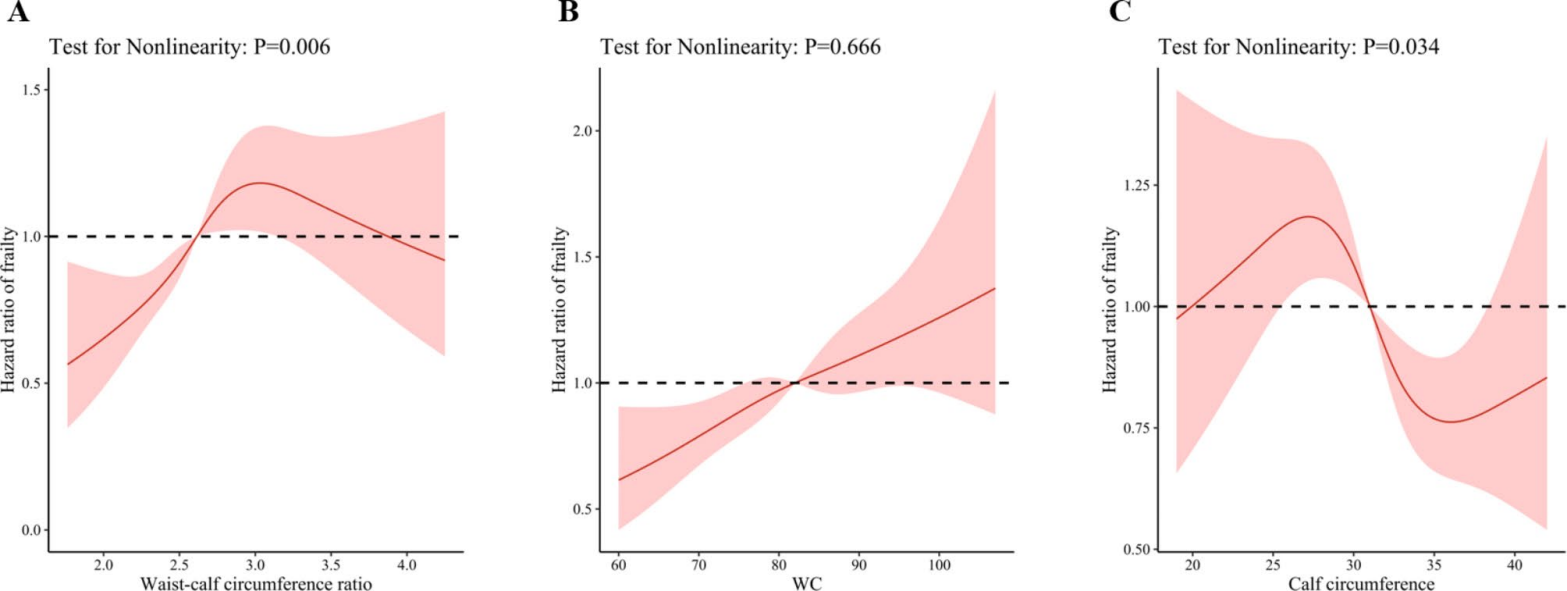
Dose-response association between anthropometric measures and frailty. Notes: Solid red lines are multivariable-adjusted hazard ratios, with shaded areas showing 95% confidence intervals derived from restricted cubic spline regressions with four knots. Reference lines for no association are indicated by dashed black bold lines at a hazard ratio of 1.0. The model was adjusted for baseline age, sex, marital status, education, residence, living arrangement, economic status, smoking status, drinking status, regular exercise status, sleep time, and body mass index, and further adjusted for calf circumference in the waist circumference model and waist circumference in calf circumference model

The mean (SD) follow-up time was 3.90 (0.51) years, with a range of 3.16 to 5.17 years. During the 9204.83 person-years of follow-up, 668 participants (28.3%) were identified as having frailty. Using restricted cubic splines, we observed that the risk of frailty decreased steadily for WCR less than 2.62 and WC less than 75.82 cm (P for non-linearity = 0.006 and 0.666, respectively) (Fig. 1). The association between CC and frailty was J-shaped. CC measurements between 25.50 and 31.00 cm were associated with an increased risk of frailty, while measurements between 31.00 and 38.42 cm were associated with a decreased risk of frailty (P for non-linearity = 0.034) (Fig. 1).

According to ROC curve analysis, the optimal cut-point values were 2.64 for WCR (sensitivity 57.8%; specificity 57.2%), 88.50 cm for WC (sensitivity 29.3%; specificity 74.6%), 31.50 cm for CC (sensitivity 60.0%; specificity 53.7%), and 21.22 kg/m^2^ for BMI (sensitivity 44.3%; specificity 61.3%) (Supplementary Fig. 2). Areas under the ROC curves (AUC) for WCR, WC, CC, and BMI were 0.592 (P < 0.001), 0.499 (P = 0.537), 0.584 (P < 0.001), and 0.525 (P = 0.972), respectively (Supplementary Fig. 2). Based on the Delong test, WCR showed a significantly higher AUC than WC (Delong test, P < 0.001) and BMI (Delong test, P < 0.001).

We categorized anthropometric measures into quintiles. Compared to participants in the first quintile, those in higher quintiles of WCR and WC had a greater likelihood of developing frailty (the fifth quintile of WCR: HR = 1.59, 95% CI 1.24–2.04, P for trend < 0.001; the fifth quintile of WC: HR = 1.69, 95% CI 1.27–2.24, P for trend < 0.001). However, those in the fourth quintile of CC had a lower likelihood of developing frailty compared to those in the first quintile (the fourth quintile: HR = 0.67, 95% CI 0.50–0.89, P for trend = 0.026) (Table 2). Similar statistically significant results were obtained when WCR, WC, and CC were categorized by the optimal cut-point (Table 2). The sensitivity analysis results were consistent with the main analyses (Supplementary Table 3).

**Table 2:** Association of anthropometric measures with incident frailty

Variables	No. Of events/total	Incidence rate ^a^	Unadjusted Model	Model 1	Model 2
			HR (95% CI)	HR (95% CI)	HR (95% CI)
Waist-calf circumference ratio					
Quintile 1 (≤ 2.36)	100 (473)	53.6	Reference	Reference	Reference
Quintile 2 (2.36–≤2.54)	106 (471)	57.8	1.18 (0.90–1.55)	1.16 (0.88–1.53)	1.19 (0.90–1.57)
Quintile 3 (2.54–≤2.69)	133 (478)	71.3	1.40 (1.08–1.81)	1.32 (1.01–1.71)	1.33 (1.03–1.73)
Quintile 4 (2.69–≤2.91)	143 (466)	79.5	1.69 (1.31–2.18)	1.50 (1.16–1.95)	1.48 (1.14–1.92)
Quintile 5 (> 2.91)	186 (471)	101.0	2.07 (1.62–2.64)	1.71 (1.33–2.20)	1.59 (1.24–2.04)
P value for trend ^b^			< 0.001	< 0.001	< 0.001
Below optimal cut-point value (< 2.64)	282 (1250)	57.6	Reference	Reference	Reference
Above optimal cut-point value (≥ 2.64)	386 (1109)	89.6	1.65 (1.42–1.93)	1.45 (1.23–1.69)	1.36 (1.16–1.59)
Waist circumference, cm					
Quintile 1 (≤ 74.00)	148 (499)	75.3	Reference	Reference	Reference
Quintile 2 (74.00–≤80.00)	160 (581)	71.0	1.07 (0.85–1.33)	1.18 (0.94–1.48)	1.22 (0.97–1.53)
Quintile 3 (80.00–≤84.80)	91 (335)	70.4	1.08 (0.83–1.41)	1.28 (0.97–1.67)	1.31 (1.00-1.72)
Quintile 4 (84.80–≤90.00)	138 (503)	70.1	1.00 (0.79–1.26)	1.35 (1.05–1.74)	1.39 (1.07–1.79)
Quintile 5 (> 90.00)	131 (441)	76.0	1.10 (0.87–1.39)	1.57 (1.19–2.07)	1.69 (1.27–2.24)
P value for trend ^b^			0.608	< 0.001	< 0.001
Below optimal cut-point value (< 88.50)	472 (1734)	69.7	Reference	Reference	Reference
Above optimal cut-point value (≥ 88.50)	196 (625)	80.4	1.20 (1.02–1.42)	1.47 (1.21–1.78)	1.50 (1.24–1.81)
Calf circumference, cm					
Quintile 1 (≤ 28.00)	207 (565)	93.6	Reference	Reference	Reference
Quintile 2 (28.00–≤30.00)	138 (436)	82.4	0.96 (0.77–1.19)	1.02 (0.82–1.27)	1.02 (0.82–1.27)
Quintile 3 (30.00–≤33.00)	157 (609)	66.6	0.77 (0.63–0.95)	0.88 (0.70–1.09)	0.89 (0.71–1.11)
Quintile 4 (33.00–≤35.00)	80 (352)	57.5	0.57 (0.44–0.73)	0.77 (0.58–1.01)	0.67 (0.50–0.89)
Quintile 5 (> 35.00)	86 (397)	54.9	0.57 (0.44–0.73)	0.82 (0.61–1.09)	0.78 (0.58–1.05)
P value for trend ^b^			< 0.001	0.068	0.026
Below optimal cut-point value (< 31.50)	401 (1184)	87.3	Reference	Reference	Reference
Above optimal cut-point value (≥ 31.50)	267 (1175)	57.9	0.62 (0.53–0.73)	0.77 (0.65–0.92)	0.73 (0.61–0.88)

*HR* hazard ratio, *CI* confidence interval

Notes: ^a^ Incidence rates per 1000 person-years

^b^ Test for trend based on the variable containing the median value for each quintile

Model 1 adjusted for baseline age, sex, marital status, education, residence, living arrangement, economic status, smoking status, drinking status, regular exercise status, sleep time, and body mass index

Model 2: further adjusted for baseline frailty index, and further adjusted for calf circumference in the waist circumference model and waist circumference in the calf circumference model

### Associations between longitudinal changes in anthropometric measures and frailty

In the 2018 wave, anthropometric measures were repeated for 2218 participants. To analyze the association between anthropometric measures and frailty risk, we conducted logistic GEE analyses for the main analyses. As shown in Table 3, increased WCR and WC were found to be associated with an elevated risk of frailty (WCR: OR = 1.74; 95% CI 1.43–2.12; WC: OR = 1.03; 95% CI 1.02–1.04) in the multivariable-adjusted model, while increased CC showed an inverse correlation with frailty (OR = 0.95; 95% CI 0.93–0.97).

**Table 3:** The associations between longitudinal changes in anthropometric measures and frailty^a^

Variables	Unadjusted model	Multivariable-adjusted model
	OR (95% CI)	OR (95% CI)
Waist-calf circumference ratio	2.25 (1.90–2.66)	1.74 (1.43–2.12)
Waist circumference, cm	1.01 (1.00-1.01)	1.03 (1.02–1.04)
Calf circumference, cm	0.92 (0.91–0.94)	0.95 (0.93–0.97)

*OR* odd ratio, *CI* confidence interval

Notes: ^a^ The generalized estimation equation was used to explore the association between longitudinal changes in anthropometric measures over time and frailty. The multivariate model was adjusted for age, sex, marital status, education, residence, living arrangement, economic status, smoking status, drinking status, regular exercise status, sleep time, and body mass index, and further adjusted for calf circumference in the waist circumference model and waist circumference in the calf circumference model

### Effect modification

**Fig. 2:**
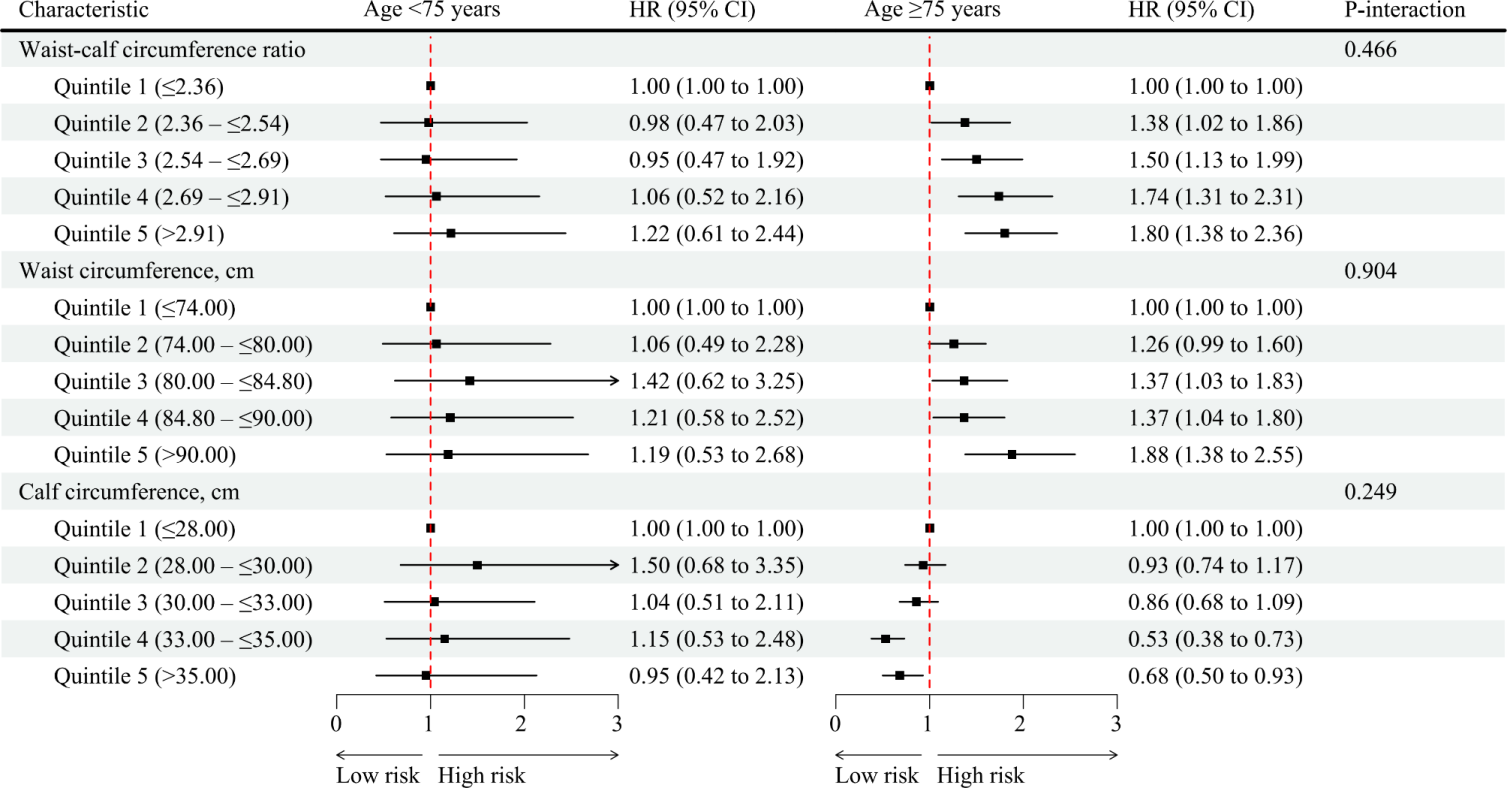
Association of anthropometric indicators with frailty stratified by age. *HR* hazard ratio, *CI* confidence interval, *WCR* waist-calf circumference ratio, *WC* waist circumference, *CC* calf circumference. Notes: The multivariate model was adjusted for baseline age, sex, marital status, education, residence, living arrangement, economic status, smoking status, drinking status, regular exercise status, sleep time, body mass index, and frailty index, and further adjusted for calf circumference in the waist circumference model, and waist circumference in the calf circumference model

In subgroup analyses, the effect sizes between WCR and WC, and CC and frailty were stronger among older adults ≥ 75 years (versus older adults < 75 years), and among women (versus men) (Fig. 2 and Supplementary Fig. 3). Furthermore, sex modified the association of WCR and frailty (P for interaction = 0.016) (Supplementary Fig. 3), while anthropometric measures and frailty were not significantly modified by other variables (Supplementary Figs. 4–6).

## Discussion

In this prospective cohort study, we investigated the relationship between anthropometric measures and the incidence of frailty in older adults. Our findings indicate that higher WCR and WC, as well as lower CC, are each significantly associated with a higher risk of frailty, independent of BMI and other potential confounders. Notably, our findings indicate that WCR exhibited a stronger association with frailty compared to WC, CC, or BMI. To the best of our knowledge, this is the first cohort study to examine the association between WCR and the risk of frailty in older people.

Our findings support previous research that has linked central obesity, as measured by WC, to an increased risk of frailty [11]. Additionally, we found that CC, which is often used as a proxy for muscle mass [23], was negatively correlated with frailty in older adults, consistent with a previous study [24]. In contrast to some prior research, we also investigated the association between longitudinal changes in WC and CC and the risk of frailty and observed consistent results. Interestingly, we used restricted cubic spline curves to examine the relationship between continuous values of CC and frailty risk and found that CC values below 25.50 cm or above 38.42 cm were not significantly associated with frailty risk. This could be due to variations in WC changes among these participants, which may have obscured the impact of muscle mass on frailty.

The impact of abdominal fat on disease risk contrasts with that of leg lean mass. Thus, understanding obesity and frailty in older adults may be better served by analyzing the distribution of body fat and lean mass rather than relying solely on BMI, WC, or CC, especially given the age-related loss of muscle mass and increase in fat mass [25]. In our study, we used the WCR to measure the imbalance between abdominal fat and calf muscle mass and found a positive correlation between WCR and frailty. Our results also found that WCR may have a stronger association with frailty. This suggests that high visceral fat and low lean body mass could contribute to frailty. However, research on the relationship between WCR and frailty in older adults is limited, which makes it challenging to compare our findings with other studies. Although the underlying mechanisms linking WCR to frailty are not fully understood, they likely involve the negative effects of central obesity on metabolic, hormonal, insulin resistance, and inflammatory pathways [26, 27]. Insulin resistance and inflammation can reduce muscle mass and strength [28, 29], potentially contributing to frailty. Muscle mass plays a vital role in physical function and mobility, making its decline a contributing factor to the development of frailty [30]. WCR is an easily measurable anthropometric indicator that can be incorporated into clinical practice at a minimal cost. This could aid in the development of intervention strategies, such as exercise and nutrition, aimed at managing the growing incidence of frailty in older adults.

One noteworthy aspect of this study is that the relationship between WCR and frailty may be affected by age and sex. Specifically, the association between WCR and frailty was found to be stronger in individuals aged over 75 years old, although the interaction term did not reach statistical significance. This may be due to the accumulation of oxidative stress and systemic inflammation resulting from long-term visceral fat accumulation and loss of lean body mass. In general, impairments in activities of daily living are more prevalent in the oldest individuals [31], which can restrict their mobility and lead to increased visceral fat accumulation [32]. Additionally, women showed a stronger association between WCR and frailty, indicating a modifying effect of sex. As women tend to have greater adipose stores than men [33], even those with a normal body mass index (BMI), their body fat percentage is similar to that of an obese male [34]. This may explain why older women are more susceptible to frailty than men with the same WCR. However, these hypotheses require further investigation with specific data to confirm the results and elucidate the underlying mechanisms.

### Strengths and limitations

We assessed the association between anthropometric measures and frailty using a prospective design and a large sample size among community old people in China. We also assessed the association between longitudinal changes in anthropometric measures over time and frailty. Trained staff measured the anthropometric variables instead of relying on self-reported data. First, our results may not apply to other populations or younger people since they were based on older adults from China. Second, the self-reporting of diseases and symptoms may result in recall bias. Finally, data from two waves of a national cohort restricted us to a four-year time horizon, so we were not able to determine impact beyond that period.

## Conclusions

Our findings indicate that WCR and WC were positively associated with frailty, while CC had a negative association. Specifically, WCR was found to have a stronger association with frailty compared to each circumference individually. These results suggest that measuring WCR could be a useful and convenient method to identify older adults who are at risk of frailty. Further research is necessary to validate and enhance the use of WCR in frailty assessment and to develop interventions that target the modifiable risk factors associated with both WCR and frailty.

### Electronic supplementary material

Below is the link to the electronic supplementary material.

Supplementary Material 1

## Data Availability

Data are from the Chinese Longitudinal Healthy Longevity Survey, which is a public, open-access repository (https://opendata.pku.edu.cn/dataverse/CHADS).

## Abbreviations

FI: frailty index
WCR: waist-calf circumference ratio
WC: waist circumference
CC: calf circumference
BMI: body mass index
GEE: generalized estimating equation
HR: hazard ratio
OR: odds ratio
CI: confidence interval
CLHLS: Chinese Longitudinal Healthy Longevity Survey
