# Supplementary material for: Is waist-calf circumference ratio associated with frailty in older adults? Findings from a cohort study
Source: BMC Geriatr. 2023 Aug 15;23:492. doi: 10.1186/s12877-023-04182-9 (PMC10426214; doi:10.1186/s12877-023-04182-9)
Supplement: Supplementary file 1 — Supplementary Material 1 [file 12877_2023_4182_MOESM1_ESM.docx]

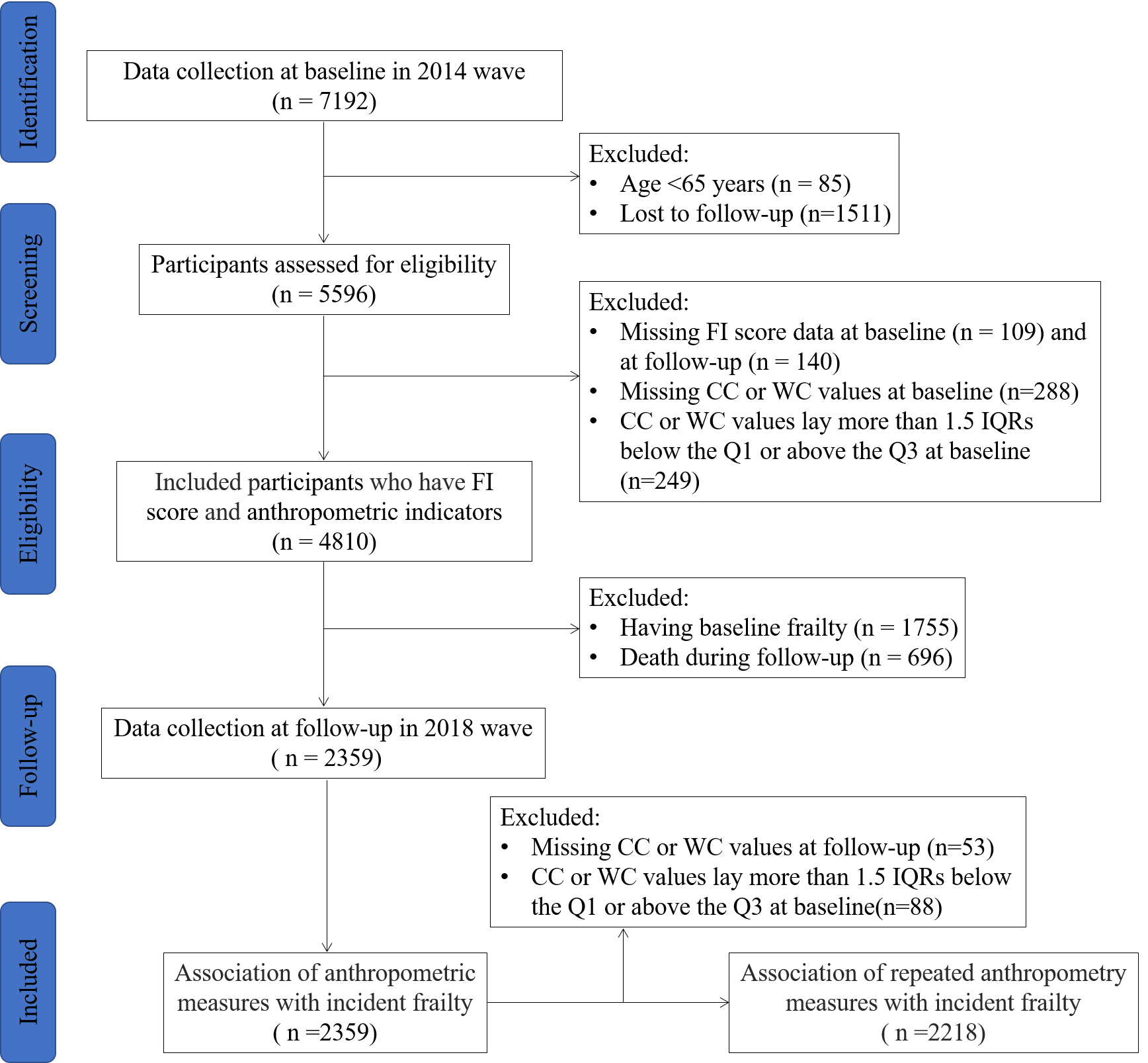


**Supplementary Figure 1.** Flowchart of the included study population. *FI* frailty index, *CC* calf circumference, *WC* waist circumference, *IQRs* interquartile ranges, *Q1* quartile 1, *Q3* quartile 3.


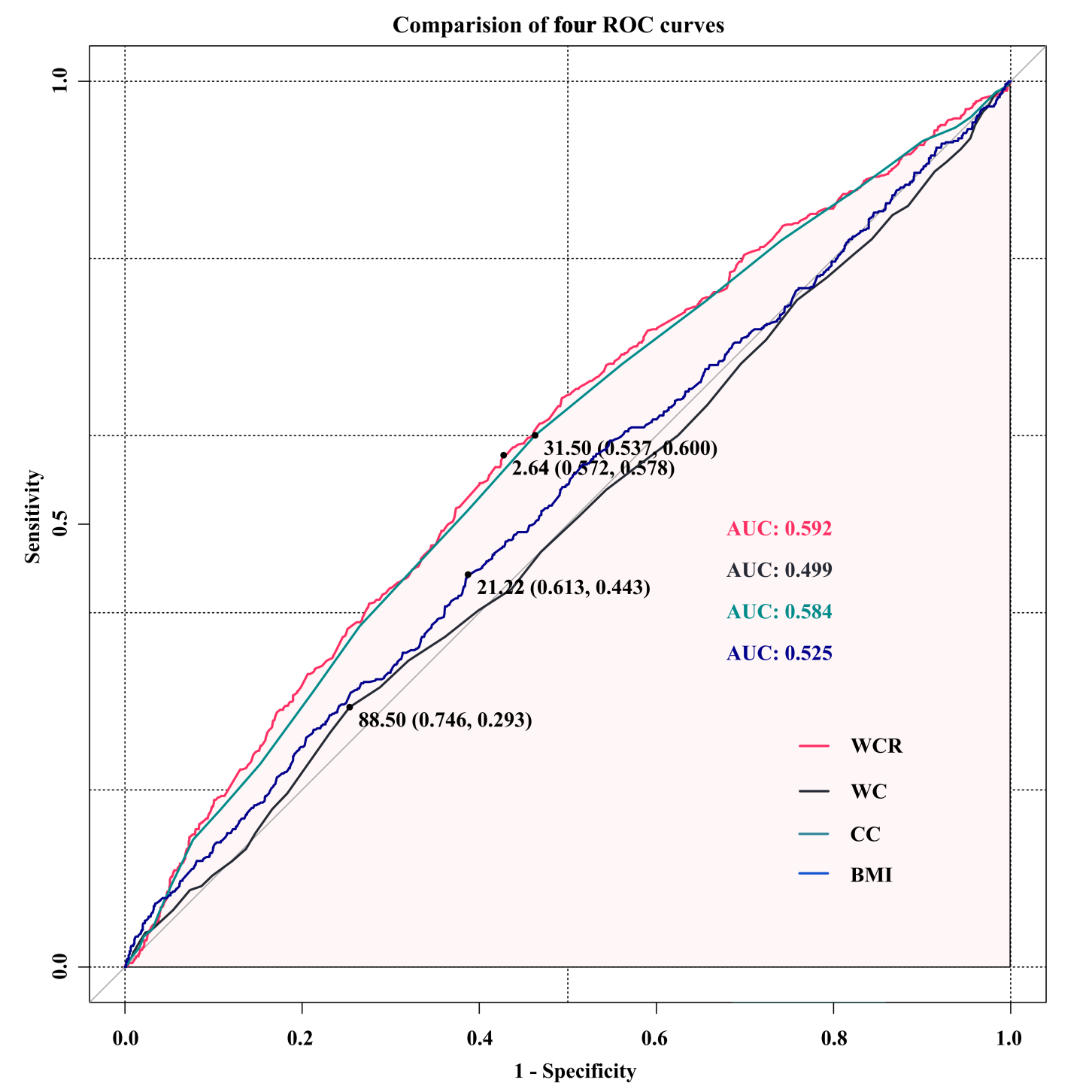


**Supplementary Figure 2** Receiver operating characteristic curves of anthropometric indicators.

*WCR* waist-calf circumference ratio, *WC* waist circumference, *CC* calf circumference, *BMI* body mass index, *AUC* Areas under the ROC curve.

**
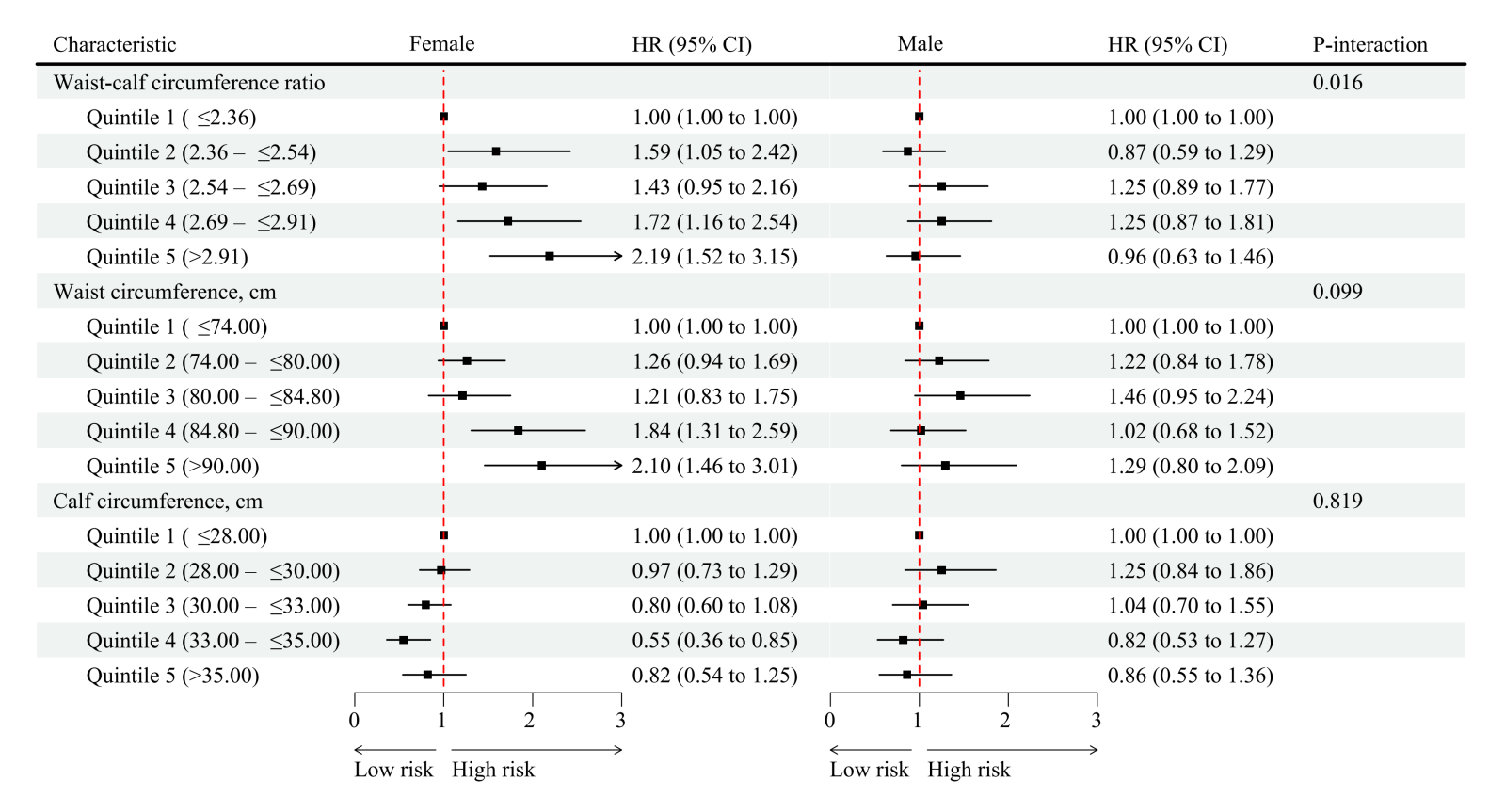
Supplementary Figure 3** Association of anthropometric indicators with frailty stratified by sex.

*HR* hazard ratio, *CI* confidence interval, *WCR* waist-calf circumference ratio, *WC* waist circumference, *CC* calf circumference.

Notes: The multivariate model was adjusted for baseline age, marital status, education, residence, living arrangement, economic status, smoking status, drinking status, regular exercise, sleep time, body mass index, and frailty index, and further adjusted for calf circumference in the waist circumference model, and waist circumference in the calf circumference model.

**
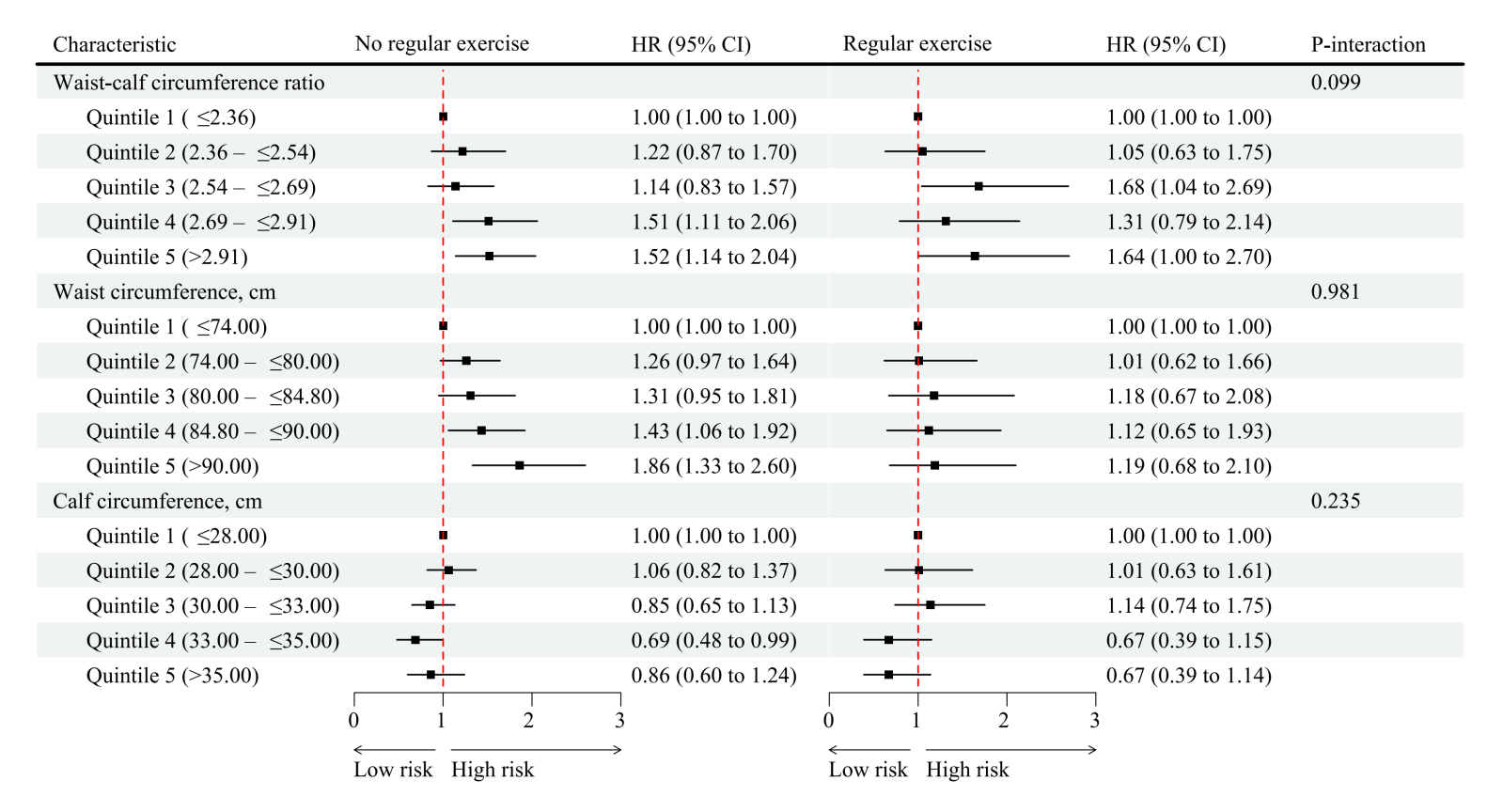
**

**Supplementary Figure 4** Association of anthropometric indicators with frailty stratified by current regular exercise status.

*HR* hazard ratio, *CI* confidence interval, *WCR* waist-calf circumference ratio, *WC* waist circumference, *CC* calf circumference.

Notes: The multivariate model was adjusted for baseline age, sex, marital status, education, residence, living arrangement, economic status, smoking status, drinking status, sleep time, body mass index, and frailty index, and further adjusted for calf circumference in the waist circumference model, and waist circumference in the calf circumference model.

**
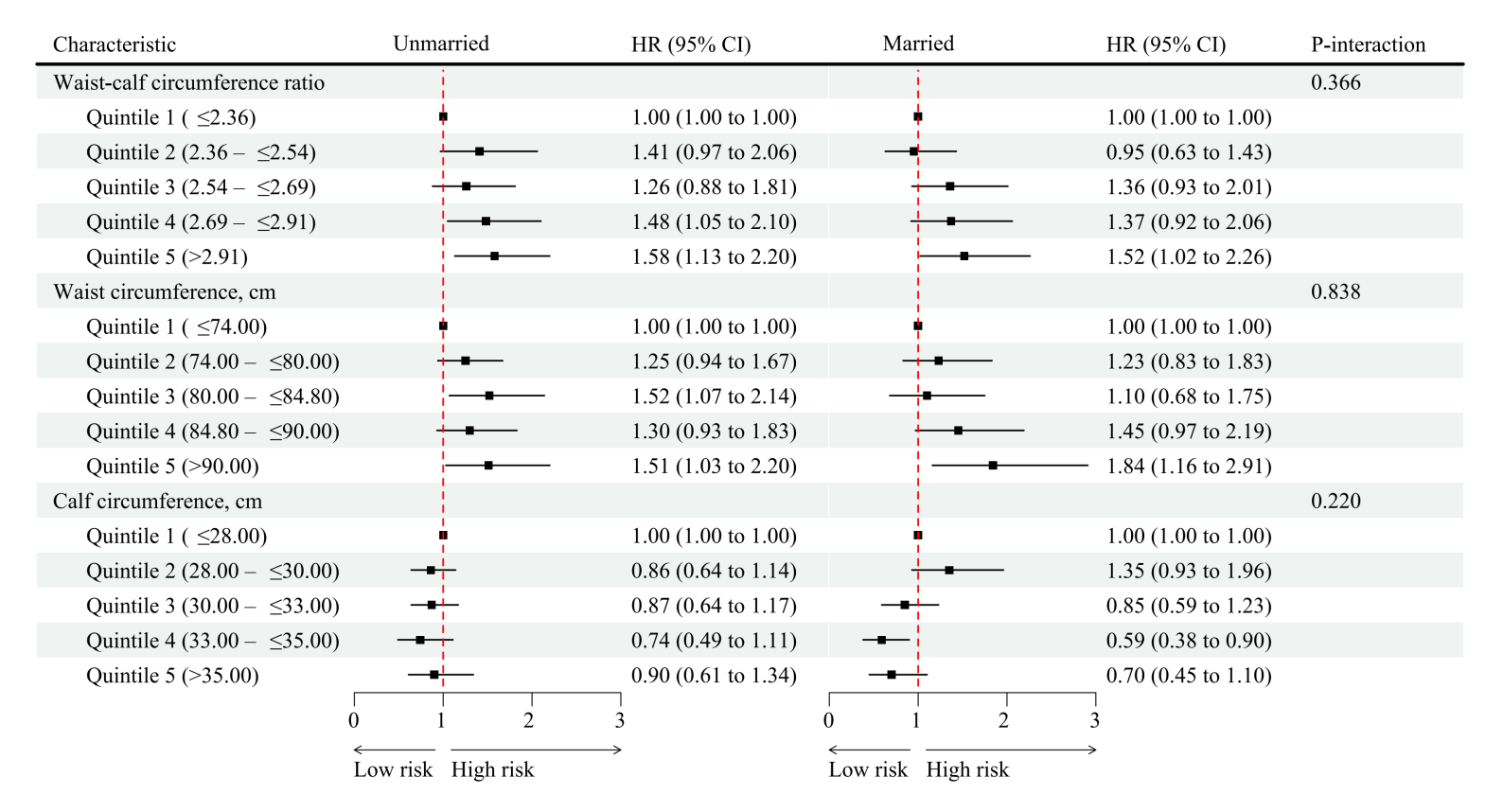
Supplementary Figure 5** Association of anthropometric indicators with frailty stratified by marital status.

*HR* hazard ratio, *CI* confidence interval, *WCR* waist-calf circumference ratio, *WC* waist circumference, *CC* calf circumference.

Notes: The multivariate model was adjusted for baseline age, sex, education, residence, living arrangement, economic status, smoking status, drinking status, regular exercise, sleep time, body mass index, and frailty index, and further adjusted for calf circumference in the waist circumference model, and waist circumference in the calf circumference model.

**
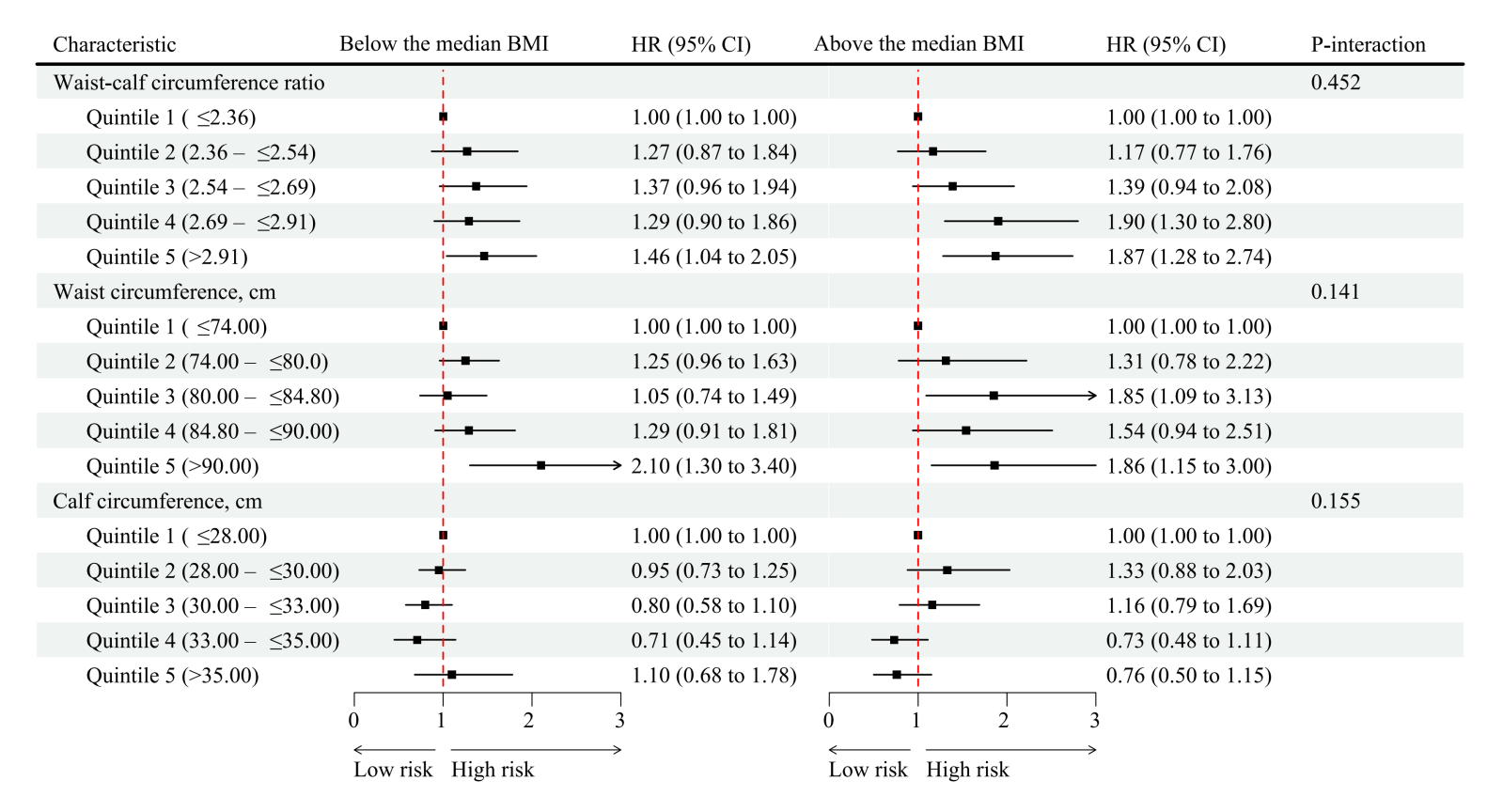
**

**Supplementary Figure 6** Association of anthropometric indicators with frailty stratified by body mass index.

*HR* hazard ratio, *CI* confidence interval, *WCR* waist-calf circumference ratio, *WC* waist circumference, *CC* calf circumference.

Notes: The multivariate model was adjusted for baseline age, sex, marital status, education, residence, living arrangement, economic status, smoking status, drinking status, regular exercise, sleep time, and frailty index, and further adjusted for calf circumference in the waist circumference model, and waist circumference in the calf circumference model.

**Supplementary Table 1**

Health variables and cut-points for the frailty index

| NO. | Items | Cut point |
| --- | --- | --- |
| 1 | Self-rated health | Very bad = 1, Bad= 0.75, So so = 0.5, Good = 0.25, Very good = 0 |
| 2 | Health worsened in the past year | Much worse = 1, A little worse = 0.75, No change = 0.5, A little better = 0.25, Much better = 0 |
| 3 | Feel fearful or anxious | Always = 1, Often =0.75, Sometimes = 0.5, Seldom = 0.25, Never = 0 |
| 4 | Feel lonely and isolated | Always = 1, Often =0.75, Sometimes = 0.5, Seldom = 0.25, Never = 0 |
| 5 | Feel useless with age | Always = 1, Often =0.75, Sometimes = 0.5, Seldom = 0.25, Never = 0 |
| 6 | Lost interest in most things like hobbies, work, or similar activities | Yes = 1, No = 0 |
| 7 | Cognitively impaired (based on the Mini-Mental State Examination) | ≤10 = 1, 11-17 = 0.75, 18-20 = 0.5, 21-24 = 0.25, ≥25 = 0 |
| 8 | ADLs: Needs assistance bathing | More than one part assistance = 1, Partial assistance = 0.5, Without assistance = 0 |
| 9 | ADLs: Needs assistance dressing | Assistance in getting clothes and getting dressed = 1, Need assistance for trying shoes = 0.5, Without assistance = 0 |
| 10 | ADLs: Needs assistance toileting | Don't use toilet = 1, Assistance in cleaning or arranging clothes = 0.5, Without assistance = 0 |
| 11 | ADLs: Needs assistance in indoor transferring | Bedridden = 1, With assistance = 0.5, Without assistance = 0 |
| 12 | ADLs: Incontinence | Incontinent = 1, Occasional accidents = 0.5, Without assistance = 0 |
| 13 | ADLs: Needs assistance eating | Need feeding = 1, With some help = 0.5, Without assistance = 0 |
| 14 | IADLs: able to visit neighbors by oneself | Unable to do so = 1, A little difficult = 0.5, Yes = 0 |
| 15 | IADLs: able to shop by oneself if necessary | Unable to do so = 1, A little difficult = 0.5, Yes = 0 |
| 16 | IADLs: able to cook meals by oneself if necessary | Unable to do so = 1, A little difficult = 0.5, Yes = 0 |
| 17 | IADLs: able to wash clothing by oneself | Unable to do so = 1, A little difficult = 0.5, Yes = 0 |
| 18 | IADLs: able to walk continuously for 1 kilometer | Unable to do so = 1, A little difficult = 0.5, Yes = 0 |
| 19 | IADLs: able to lift a weight of 5 kg (such as a heavy bag of groceries) | Unable to do so = 1, A little difficult = 0.5, Yes = 0 |
| 20 | IADLs: able to continuously crouch and stand up three times | Unable to do so = 1, A little difficult = 0.5, Yes = 0 |
| 21 | IADLs: able to use public transportation | Unable to do so = 1, A little difficult = 0.5, Yes = 0 |
| 22 | Able to use chopsticks to eat | No = 1, Yes = 0 |
| 23 | Able to put a hand behind the neck | Neither hand = 1, Right or left hand only = 0.5, Both hands = 0 |
| 24 | Able to put a hand behind the lower back | Neither hand = 1, Right or left hand only = 0.5, Both hands = 0 |
| 25 | Able to raise arm upright | Neither hand = 1, Right or left hand only = 0.5, Both hands = 0 |
| 26 | Able to stand up from sitting in a chair | No = 1, Yes, using hands = 0.5, Yes, without using hands = 0 |
| 27 | Able to pick up a book from the floor | No = 1, Yes, sitting = 0.5, Yes, standing = 0 |
| 28 | Vision loss | Can't see or blind = 1, Can see only = 0.5, Can see and distinguish = 0 |
| 29 | Hearing loss | Yes = 1, No = 0 |
| 30 | Number of serious illnesses in the past 2 years | Two or more illnesses or bedridden = 2, one illness = 1, No = 0 |
| 31 | Suffering from hypertension | Yes = 1, No = 0 |
| 32 | Suffering from diabetes | Yes = 1, No = 0 |
| 33 | Suffering from heart disease | Yes = 1, No = 0 |
| 34 | Suffering from stroke/cerebrovascular disease | Yes = 1, No = 0 |
| 35 | Suffering from bronchitis, emphysema, asthma, or pneumonia | Yes = 1, No = 0 |
| 36 | Suffering from tuberculosis | Yes = 1, No = 0 |
| 37 | Suffering from cancer | Yes = 1, No = 0 |
| 38 | Suffering from Parkinson's disease | Yes = 1, No = 0 |
| 39 | Suffering from arthritis | Yes = 1, No = 0 |
| 40 | Poor interviewer-rated health | Yes = 1, No = 0 |

*ADLs* activities of daily, *IADLs* instrumental activities of daily living.

**Supplementary Table 2**

The numbers (percentage) of the missing variables ^a^

| Characteristics | Number (%) with missing data |  |
| --- | --- | --- |
| Living arrangement | 24 (1.0) |  |
| Marital status | 32 (1.4) |  |
| Education | 25 (1.1) |  |
| Smoking status | 9 (0.4) |  |
| Drinking status | 20 (0.8) |  |
| Regular exercise status | 51 (2.2) |  |
| Economic status | 20 (0.8) |  |
| Sleep time | 12 (0.5) |  |
| Body Mass Index | 37 (1.6) |  |

Notes: ^a^ List only the variables with missing data.

**Supplementary Table 3**

Sensitive analysis of the association between anthropometric measures and frailty after excluding participants with missing covariate data

| Variables | Unadjusted model | Model 1 | Model 2 |
| --- | --- | --- | --- |
|  | HR (95% CI) | HR (95% CI) | HR (95% CI) |
| Waist-calf circumference ratio | | | |
| Quintile 1 (≤2.36) | Reference | Reference | Reference |
| Quintile 2 (2.36–≤2.54) | 1.21 (0.92-1.61) | 1.17 (0.88-1.55) | 1.20 (0.91-1.59) |
| Quintile 3 (2.54–≤2.69) | 1.37 (1.05-1.79) | 1.29 (0.98-1.69) | 1.31 (1.00-1.72) |
| Quintile 4 (2.69–≤2.91) | 1.69 (1.29-2.20) | 1.50 (1.14-1.96) | 1.49 (1.13-1.95) |
| Quintile 5 (>2.91) | 1.97 (1.52-2.54) | 1.63 (1.25-2.12) | 1.54 (1.18-2.00) |
| P for trend ^a^ | <0.001 | <0.001 | <0.001 |
| Below optimal cut-point value (<2.64) | Reference | Reference | Reference |
| Above optimal cut-point value (≥2.64) | 1.59 (1.36-1.87) | 1.40 (1.19-1.65) | 1.33 (1.13-1.57) |
| Waist circumference, cm | | | |
| Quintile 1 (≤74.00) | Reference | Reference | Reference |
| Quintile 2 (74.00–≤80.00) | 1.08 (0.85-1.36) | 1.18 (0.93-1.50) | 1.22 (0.96-1.56) |
| Quintile 3 (80.00–≤84.80) | 1.07 (0.81-1.42) | 1.25 (0.94-1.67) | 1.27 (0.95-1.70) |
| Quintile 4 (84.80–≤90.00) | 1.07 (0.84-1.37) | 1.43 (1.10-1.86) | 1.48 (1.13-1.93) |
| Quintile 5 (>90.00) | 1.12 (0.88-1.44) | 1.55 (1.16-2.08) | 1.66 (1.23-2.24) |
| P for trend ^a^ | 0.392 | 0.001 | <0.001 |
| Below optimal cut-point value (<88.50) | Reference | Reference | Reference |
| Above optimal cut-point value (≥88.50) | 1.25 (1.05-1.49) | 1.52 (1.24-1.86) | 1.54 (1.26-1.89) |
| Calf circumference, cm | | | |
| Quintile 1 (≤28.00) | Reference | Reference | Reference |
| Quintile 2 (28.00–≤30.00) | 1.00 (0.79-1.26) | 1.04 (0.82-1.31) | 1.02 (0.80-1.29) |
| Quintile 3 (30.00–≤33.00) | 0.81 (0.65-1.01) | 0.92 (0.73-1.17) | 0.92 (0.72-1.17) |
| Quintile 4 (33.00–≤35.00) | 0.61 (0.46-0.80) | 0.81 (0.60-1.08) | 0.69 (0.51-0.93) |
| Quintile 5 (>35.00) | 0.61 (0.47-0.79) | 0.84 (0.62-1.13) | 0.79 (0.58-1.07) |
| P for trend ^a^ | <0.001 | 0.150 | 0.051 |
| Below optimal cut-point value (<31.50) | Reference | Reference | Reference |
| Above optimal cut-point value (≥31.50) | 0.66 (0.56-0.78) | 0.81 (0.67-0.97) | 0.77 (0.63-0.92) |

*HR* hazard ratio, *CI* confidence interval.

Notes: ^a^ Test for trend based on the variable containing the median value for each quintile.

Model 1: adjusted for baseline age, sex, marital status, education, residence, living arrangement, economic status, smoking status, drinking status, regular exercise status, sleep time, and body mass index.

Model 2: further adjusted for baseline frailty index, and further adjusted for calf circumference in the waist circumference model and waist circumference in the calf circumference model.
